# Assessment of the Environmental Impacts of a Localized Food System and Food Waste Reduction in a Water-Scarce Region Using Diet Optimization Models

**DOI:** 10.3390/ijerph20105890

**Published:** 2023-05-20

**Authors:** Felix Haifeng Liao, Robert Heinse, Darin Saul, Soren Newman, Li Huang, Colette DePhelps, Steven Peterson

**Affiliations:** 1 Department of Earth and Spatial Sciences, University of Idaho, Moscow, ID 83844, USA; hliao@uidaho.edu; 2 Department of Soil and Water Systems, University of Idaho, Moscow, ID 83844, USA; rheinse@uidaho.edu; 3 Arrowleaf Consulting, Walla Walla, WA 99362, USA; darin@arrowleafgroup.com (D.S.); soren@arrowleafgroup.com (S.N.); 4 Institute for Modeling Collaboration and Innovation, University of Idaho, Moscow, ID 83844, USA; 5 Northern District Extension, University of Idaho, Moscow, ID 83844, USA; cdephelps@uidaho.edu; 6 College of Business and Economics, University of Idaho, Moscow, ID 83844, USA; stevenp@uidaho.edu

**Keywords:** local food, fruits and vegetables, diet optimization, food loss and waste, high-resolution water footprints, Inland Northwest

## Abstract

Despite growing interest in fresh local produce across the United States, scaling up local agricultural development might impose new environmental pressures on increasingly scarce water and land resources in specific localities. Drawing upon the case of the Palouse of the US Inland Northwest, this study evaluates land and water footprints of local foods along with food waste reduction in a water-scarce region. We used both non-robust and robust diet-optimization techniques to estimate the minimum amounts of irrigation water necessary to grow foods locally and to satisfy the local population’s caloric or nutrition needs. Our modeling results indicate that, on an annual basis, an increase of less than 5% of the current freshwater withdrawal on the Palouse could satisfy 10% of the local population’s aspirational demand for locally grown food products, while more than 35% of local foods (by mass) may be wasted. Furthermore, reducing food waste by 50% could simultaneously reduce water use by up to 24%, cropland use by 13%, and pastureland use by 20%. Our findings not only provide intriguing information for access to local food but could also be used to stimulate new efforts to increase consumers’ and retailers’ awareness of environmental benefits associated with food waste reduction.

## 1. Introduction

Largely driven by increased consumer demand for fresh produce and a desire for locally sourced food, local or regional food systems have flourished near many US cities and towns. In 2020, over 147,000 farms, or 7.4% of American farms, produced and marketed their products locally [1]. Meanwhile, there have been calls for *scaling up* local food production, or developing an alternative food network [2,3], which represents the desire “to regionally distribute food and to sell into more mainstream grocery and retail venues” p. 504 [4]. This concern gained renewed attention amid the COVID-19 pandemic since the resulting supply chain shortages have brought unprecedented challenges to conventional food systems [5,6], and there has been an increased interest in healthier diets and lifestyles during the post-pandemic era [7]. In practice, scaling up is often associated with strategies that aim to strengthen the ability of small- and medium-sized farms to meet growing demand. For example, many communities are increasingly interested in supporting the development of food hubs and community-supported agriculture (CSA) [8,9]. In part, scaling up also means simultaneously increasing the production volume distributed and improving the economic efficiency of regional food systems (e.g., through food waste reduction or food recovery) [4,10]. However, as Peters et al. [11] p. 125 argued, the current debate on local food has not reached full consensus on two key questions: (1) *“do ‘local foods’ offer real ecological, economic, and social benefits?”* and (2) *“can ‘local food’ move beyond the niche markets and supply a significant share of food demand?*”

Previous studies in this area have applied geospatial and environmental impact assessment methods, such as foodshed analysis and life cycle assessment (LCA), to gauge the local agricultural capacity and potential for food localization and self-sufficiency [12,13,14,15,16,17,18]. Recent work has also been carried out to better understand how local populations could shift toward a healthier diet whilst minimizing environmental impacts with respect to land, water, energy, and greenhouse gas emissions [19,20,21,22]. For example, by using robust optimization techniques, a recent case study in Chicago suggested that the local population’s nutritional needs can be met by growing foods on farmland within 205–220 km of the city center, and including urban agriculture would reduce the radii to 115–130 km [23]. However, drawing upon the case of Santa Barbara County (CA), Cleveland et al. [19] found that if fruit and vegetable consumption could be 100% localized, it would only reduce greenhouse gas emissions from the agrifood system by less than 1% and not necessarily generate substantial nutritional benefits. Additionally, there have been indications that localizing food production and consumption could increase the potential for sustainability and circularity of regional food systems through the recycling of energy, water, and nutrients [24,25].

It should be noted that previous studies that link food system sustainability with resource capacity have been focused on a limited set of indicators and typically do not consider food waste and loss and water use impacts, while agriculture is the largest user of freshwater and is responsible for 90% of global consumptive freshwater use [26]. Reducing food waste is also considered an important element of the United Nation’s (UN) sustainable development goal [27], being one of the key themes at the UN 2021 food systems summit [28]. Focusing on the complex relationship between diet quality, food waste, and environmental sustainability, Conrad et al. [29] found that US consumers wasted nearly 1 pound or 422 g of food every day on average, and shifting toward a higher-quality diet might result in increased water footprints of food consumption. Heller et al. [30] also found a fivefold variation between the highest and lowest quantile of diets with respect to water use impacts among individual diets in the U.S. Mekonnen and Fulton [31] provided evidence that reducing food waste would be the most efficient way to reduce the water footprint of food consumption. Read et al. [32] found that food processing, food services (restaurants), and households are the three most-promising areas for food waste intervention and that reducing food waste could generate substantial environmental benefits with respect to greenhouse emissions and energy use. Read et al. [33] further suggested that simply reducing food waste would benefit global biodiversity more than a dietary change for all Americans. In short, although recent studies have documented the environmental implications of dietary shifts, in conjunction with food waste reduction, empirical evidence, e.g., [33,34], is mostly focused on making a national-scale impact. The environmental impacts of a healthier diet and reducing food waste for regional food systems have rarely been tested. Furthermore, allocating natural resources (e.g., freshwater) for local foods or fresh produce would also affect the regional-level resource availability for other competing uses, and the effect of *scaling up* may also differ from one category of natural resources to another [35]. Additional work is necessary to better understand these trade-offs tailored to local environmental issues such as water scarcity amid scaling up.

In this work, we present a case study of Whitman and Latah counties on the Palouse region of the US Inland Northwest. The case represents a key environmental challenge, namely, water scarcity that many regions face amid local and regional food system development [30]. We have three main objectives. First, we evaluate to what degree *scaling up* local agricultural development, especially through increasing the consumption and production capacity of local food, would have an impact on the region’s key environmental and agricultural resources, including irrigation water, green or rainwater, cropland, and pastureland. Second, we gauge the potential environmental pressures arising from scaling up by comparing its impact with current resource usage of other agricultural and non-agricultural sectors. Third, we model the possible environmental benefits of reducing food waste. Additionally, a series of robust (uncertain irrigation water requirements) and non-robust (average irrigation water requirements) diet optimization models were formulated to account for a pathway towards a more diverse and healthier diet in local communities, especially by increasing food diversity and consumption of fruits and vegetables. The application of these cutting-edge optimization techniques allows us to test scenarios that explicitly address the trade-offs between scaling up, reducing food waste, and lowering adverse impacts on the environment. Additionally, the aim of this case study is to showcase how a local area can utilize publicly available demographic data and dietary guidelines provided by the United States Department of Agriculture (USDA) alongside high-resolution environmental-footprint and crop-production metrics to assess the potential for expanding agricultural production and to direct local food system planning.

## 2. Materials and Methods

### 2.1. Study Region

**Figure 1:**
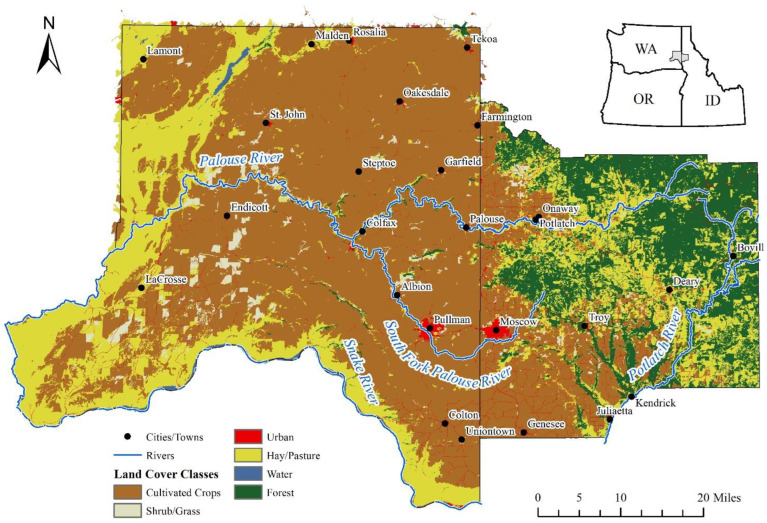
Spatial distribution of cropland and population settlements in the Palouse region (Whiteman, WA, and Latah, ID) of Inland Northwest.

The Palouse region of the Inland Northwest is one of the leading dryland wheat farming regions, producing one of the highest wheat yields in the world [36]. The area is sparsely populated, and the major population centers are the two college towns of Moscow in Idaho and Pullman in Washington (Figure 1). In 2020, Whitman and Latah counties totaled 87,490 residents [37]. According to the 2017 and 2012 United State Department of Agriculture (USDA) Census of Agriculture, there were around 2000 farms averaging over 1000 acres in Whitman County, WA, and 300 acres in Latah County, ID (Table 1) [38,39].

Like other farming areas near US cities and towns, the number and variety of local food initiatives in the Palouse region have experienced a rapid increase since the 1990s [36]. For example, the Palouse-Clearwater Food Coalition was founded in 2011 to strengthen the health and vibrancy of the local food system by increasing the production, distribution, and consumption of local agricultural products [40]. Recent survey data from local restaurants or grocery stores and direct markets, such as the farmers market, documented growing demand for local fruits and vegetables [9,41]. According to the 2012 and 2017 Censuses of Agriculture, the number of local food growers in Latah (ID) and Whitman (WA) counties increased from 87 to 125 or by 40% during the period of 2012–2017, although on average the sizes of local vegetable and fruit growers remained much smaller than the statewide averages in Idaho and Washington. Revenue derived from direct market sales also increased by 34% during the same period, even though the average sales of fruit and vegetable farms remained lower than the statewide averages (Table 1). With respect to land use, the percentage of irrigated acres in total farmland was less than 1% in both Whitman and Latah counties as compared to 23% and 58% in Washington and Idaho, respectively (Table 1), reflecting the fact that the study region is a predominately dryland agricultural area.

Due to the prominence of dryland farming systems in the Palouse area, water resources in the region are mainly used for domestic or municipal purposes (Table 2). Groundwater is the primary source of water in the region. Two aquifers, the Wanapum and the Grande Ronde, supply water for municipal, commercial, university, and residential uses [42]. The Wanapum aquifer is the primary water source for rural areas in Latah County and some areas of Whitman County while approximately 65 to 70% of the city of Moscow’s drinking water comes from the Grande Ronde aquifer [43]. In 2010, the USGS water estimation project reported that the two-county study region used about on average 73.5 thousand m^3^/day of freshwater [44], of which surface water only accounted for 13%.

However, groundwater resources have been declining on the Palouse. Led by a local organization called the Palouse Basin Aquifer Committee (PBAC), considerable attention has been paid to the scientific uncertainty of water levels in wells and water conservation on the Palouse [45]. The inter-jurisdictional nature of water governance further complicates groundwater uses. Specifically, in the state of Washington, individual wells pumping for domestic use of water not exceeding 5000 gallons per day or 141.5 m^3^/day are not required to report the volume pumped to the state, whereas the counterpart of this requirement for Idaho is 13,000 gallons per day (or 368 m^3^/day) [42]. As water levels in the wells have been dropping by 0.30–0.45 m annually in the region over the past century [46], any proposal to increase water use, such as scaling up local agriculture and small-acreage farming, may face scrutiny.

### 2.2. Environmental Footprints of Local Foods

To estimate the environmental footprints of local foods, the present study draws upon datasets of water footprint estimates and crop yield records for land-use footprints. As shown in Table 3, the selection of crops is based on their prevalence in the US food system taking into account crops that have been historically grown and are popular on the Palouse, such as lentils, chickpeas, and cherries. We also accounted for seasonal food availability and storage and processing through preservation methods (e.g., frozen or canning), following previous studies e.g., [47].

Both green or rainfall water and blue or irrigation water footprints were compiled from Meknonnen and Hoekstra (2011) and from the Water Footprint Network (WFN) [35,49]. The WFN provides high-resolution county- or state- as well as crop-level irrigation water-use estimates (m^3^/yr) that are modelled estimates at a spatial resolution of a 5 × 5 arcmin grid [48,49]. It should also be noted that the modeling results from WFN have accounted for local soil and meteorological conditions and considered the evapotranspiration (ET) requirements of each crop, so the estimates are conservative due to underestimation of other consumptive water uses such as frost protection and field preparation; however, these uses are small compared to ET requirements [48]. Next, we calculated water footprints (m^3^/1000 kg) for the Palouse region by dividing the modeled water use estimates per crop area (m^3^/ha/yr.) by the county- or state- (when the county-level yield estimate was not available) level average crop yield records (1000 kg/ha) obtained from the 2017 USDA Agricultural Census [38]. We further approximated uncertainties in water use or irrigation water requirements by dividing the modeled water consumption per crop area (m^3^/ha/yr) for each crop by its yield records from 2008 to 2019, which gives the water consumption per ton of production (m^3^/ton) and the estimates of maximum or minimum water footprints (see Table 3).

For non-crop food or animal products, we used public datasets from WFN, which include national-, state-, and county-level water footprints (WF), and calculated the production-weighted average irrigation or blue WFs using the county’s inventory data [39]. Specific animal products include the following: dairy cows, beef, and other cattle; hogs and pigs; laying hens (conversion to eggs); and broilers, chickens and turkeys [48]. For livestock production, the maximum and minimum are estimated by adjusting the animal water-use coefficients of each state to match the first and third quantile coefficients nationwide [48], and we obtained these estimates from the WFN.

With respect to land requirements, we gathered crop yield records from the USDA NASS quick stats database and the 2017 Census of Agriculture [38,39]. Where possible, yield data at the county level were used. However, state- or national-level data were used when county yield data were not available. The amount of land used for meat and dairy products was estimated by following Costello et al. [50], Dundar et al. [51], and Liao et al. [15].

### 2.3. Objectives, Constraints, and Uncertainty

**Figure 2:**
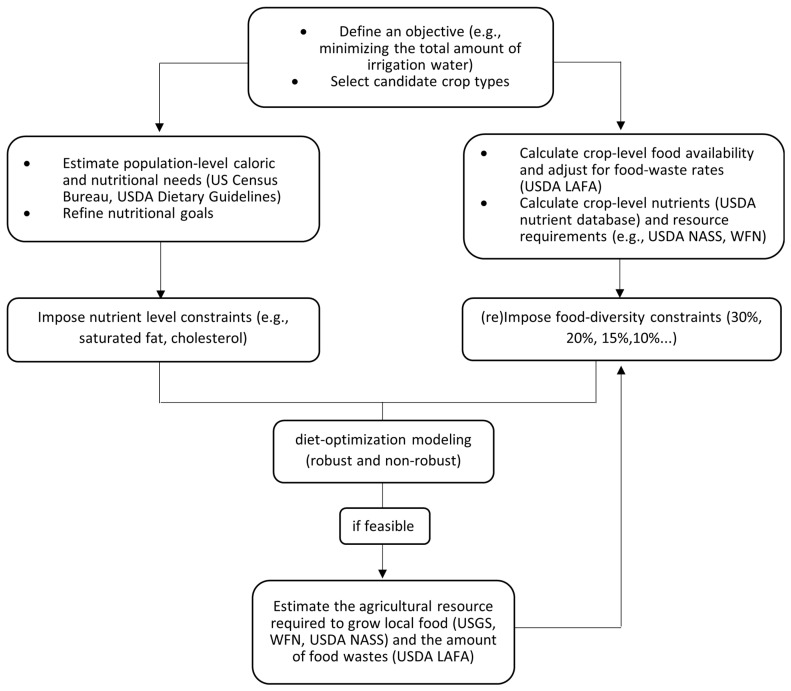
Flowchart of data collection and the logic of optimization modeling (robust and non-robust) to determine the minimal feasible amount of irrigation water, required to meet nutritional needs of the local population.

The nutrient requirements of the population segmented by age and sex were estimated using the daily food nutrient requirements recommended by the USDA Center for Nutrition Policy and Promotion, in the 2015–2020 *Dietary Guidelines for Americans* [52]. To ensure that the optimization solutions aligned with a healthier diet, the model used the USDA dietary guidelines instead of relying on actual consumption patterns [23]. The USDA recommended requirements were also chosen because diet optimization models could produce more conservative results that are suitable for assessing water use impacts associated with increased fruit and vegetable consumption [53]. The nutritional information for each food type included in the analysis was obtained from the USDA national nutrient database [54]. A list of 27 nutrients and total amounts for each food group or food type included in the analysis are available on request. Figure 2 presents an overview of datasets and model specifications. Data on population-level nutrient requirements and food waste rates and water or other environmental footprints of different food types were inputs of a set of robust (varying irrigation requirements) and non-robust (average irrigation requirements) optimization models. In this framework, users of the model first define an optimization objective and then specify the types of food crops produced as constrained by a set of minimum nutritional requirements of the population according to the daily recommended intake rates from the USDA. The second set of constraints we imposed were the maximum allowable amounts for nutrients that are often correlated with negative health outcomes (e.g., saturated fat, cholesterol). The third group of constraints refer to the maximum share of total production associated with the most-produced single food type (by weight). Hence, increasing food diversity levels could represent a pathway toward a healthier and a more desirable diet solution.

In this study, the primary objective is to minimize the use of blue or irrigation water in the Palouse region while satisfying the region’s theoretical nutritional demand (Equation (1)).

(1) $$\min\underset{i\in I}{\sum }w_{i}x_{i}$$

where i represents the set of food types, $x_{i}$ denotes the amount of food type i in kg to be produced, and $w_{i}$ denotes the amount of irrigation water in m^3^ required to produce 1000 kg of food type i.

The following constraints guarantee that the population’s nutritional or caloric requirements are satisfied:

(2) $$\underset{i\in I}{\sum }n_{ij}x_{ij}\geq \;\beta_{j}\;\;\;\;\forall \;j\;\in J$$

(3) $$\underset{i\in I}{\sum }t_{is}x_{i}\leq \;\theta_{s}\;\;\;\;\forall \;s\;\in S$$

where *j* denotes the set of nutrients, $n_{ij}$ represents amount of nutrient *j* obtained from 1 kg of food type i, and $\beta_{j}$ refers to the aggregate minimum required amount of food or nutrient *j*; $\theta_{s}$ denotes the population-level aggregate maximum allowable amount of the selected nutrient *s*.

In addition, uncertainties or variations in irrigation requirements were accounted for by replacing the parameter $w_{i}$ in the objective function (1) with the following uncertain sets (see Equation (4)). The revised objective function (4) considers the values between the lower bound or minimum and the upper bound or maximum water-footprint estimates (Table 3). The robust optimization or RO models assume that uncertain parameters reside in the uncertain set of possible outcomes, using ellipsoidal uncertainty set approach:

(4) $$\min\underset{i\in I}{\sum }{w_{i}}^{\prime }*x_{i}\;\;\;\;\;\;\;\;\;{w_{i}}^{\prime }=w_{i}\;\pm \;p_{i}{\hat{w}}_{i}$$

where $w_{i}$ is the midpoint of the range of possible values of blue water required to produce 1000 kg of food type i.; ${\hat{w}}_{i}$. shows the range half-width; $p_{i}$ is our measure of uncertainty and is constrained by $\sqrt{\sum p_{i}{}^{2}\leq 1.}$ Accordingly, the required irrigation water lies in between $\left[w_{i}-{\hat{w}}_{i,\;}\;w_{i}+{\hat{w}}_{i}\right]$ such that ${w_{i}}^{\prime }=w_{i}+p_{i}{\hat{w}}_{i}$ is the worst-case scenario occurring within the uncertain set. RSOME, an open-source Python package with the Gurobi solver, was utilized for all optimization exercises [55]. Optimal solutions may differ when uncertainty in water footprints is and is not accounted for, which is denoted as a ‘robust’ solution or a ‘non-robust’ solution, respectively.

Next, the models further include constraints of ‘food diversity levels’ (Equation (5)):

(5) $$\frac{x_{i}}{\sum_{i^{\prime \in I}}^{}{x_{i}}^{\prime }}\leq \;y\;\;\;\;\;\forall \;i\;\in I$$

where y is the upper bound for the percentage of the diet composed of food type i, and constraint (5) aims at increasing the diversity of diets or food types while satisfying the nutritional and caloric requirements of the population [23,51].

### 2.4. Alternative Scenario on Food Waste Reduction

As mentioned in the previous sections, our modeling was firstly focused on a food production scenario that aims to minimize the amount of irrigation water necessary to meet the region’s food demand or nutritional needs, and to estimate associated environmental impacts of green or rainwater, cropland, and pastureland. We further address the potential effect of food waste reduction by exploring an alternative scenario in which we assumed a 50% reduction in avoidable food waste (excluding the edible portion), following the goal proposed by USDA and the EPA to reduce food loss and waste by 2030 [56].

Regarding food availability data and food waste estimates, we retrieved national-level food waste statistics from the USDA Loss Adjusted Food Availability (LAFA) database [57]. Specifically, the LAFA database provides estimates of both food waste and edible portions of each food selected in our models [57,58]. The database also differentiates between retail-level and consumer-level food waste, although it does not include losses from primary (on-farm) food production. Among the selected twenty food types, fruits and vegetables, including both fresh and preserved, are estimated to be lost or wasted (by mass) at a rate of 50% on average, followed by eggs/dairy products (35.0%), grains (34.5%), and meat/poultry (32.3%). Following others [32], variation in the cost of food waste reduction is not considered, and we did not account for specific intervention approaches at different stages of the food supply chain from farm to fork [59]. The overarching goal was to quantify the potential benefits of food waste reduction for the environment in the context of scaling up local agriculture and increasing access to local produce.

## 3. Results

### 3.1. Estimated Amount of Food to Be Grown Locally and Its Resource Requirements

**Figure 3:**
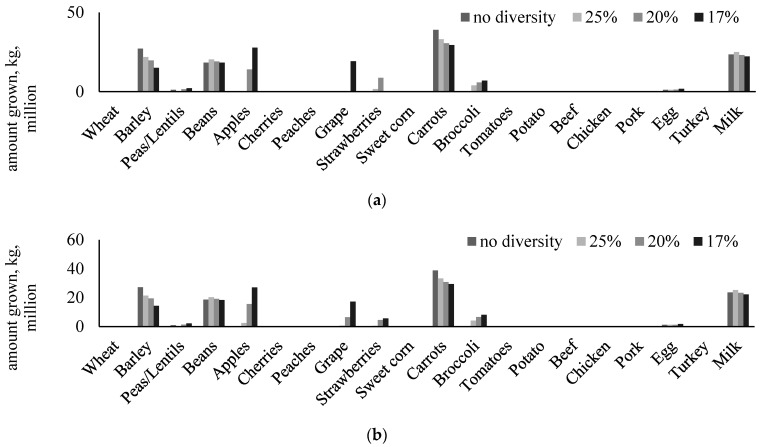
(**a**) Non-robust and (**b**) robust optimal selection of food items to meet the population’s nutritional needs while minimizing the use of irrigation water, scenarios with different food diversity levels.

With the primary objective of minimizing the use of irrigation water, our models are able to derive a solution that satisfies the nutritional needs in the Palouse area with only seven food types selected, namely barley, peas/lentils, beans, carrots, broccoli, eggs, and milk. The amount of each food item produced for the solution is shown in Figure 3. In this case, no single food type could account for more than 33% of food production by weight.

As described in the previous section, to increase the diversity of food types in the solution diet, we imposed a diversity constraint (see Equation (5)). Based on the food diversity component, the model was further instructed to favor different levels of diversity in food types and smaller values of food diversity levels indicate greater diversity in the modeling or solution diet. We were able to obtain a solution in which no single food type could account for more than 17% of the total food produced by weight for both robust and non-robust models. The results of different levels of food diversity, namely, no diversity (i.e., larger than 30%), 25%, 20%, and 17%, are shown in Figure 3, which also represent a path towards a more diverse, desirable, and healthier diet mostly by increasing the consumption of fruits and vegetables (e.g., apples, grapes, strawberries, broccoli, etc.).

Solutions derived from the RO model that account for uncertainty in the irrigation water footprint are largely identical to the results of the non-RO model, which is different from studies that aimed to minimize cropland use for local food production, e.g., [51]. However, as illustrated in Figure 3, increasing food diversity of the modeling diet, or achieving a healthier and desirable diet, would lead to increased amounts of local foods needed, especially in the category of fruits and vegetables. As mentioned previously, we were only able to obtain a solution that had no single food type that can account for more than 17% of foods grown locally. Under this scenario, the solution derived from the non-RO model utilized nine food types (i.e., barley, peas/lentils, beans, apples, grapes, carrots, broccoli, eggs, and milk), whereas the RO model utilized 11 food types with the addition of strawberries (4% of total weight) and beef (less than 1% in total weight, Figure 3).

**Figure 4:**
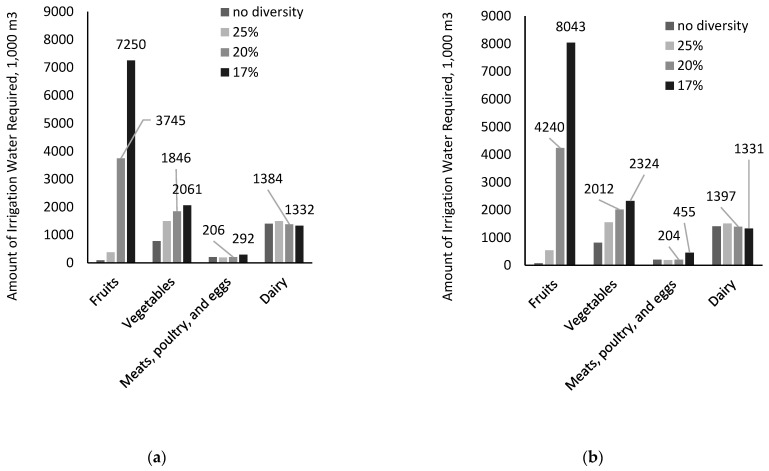
Annual minimum amounts of irrigation water in the localized food system; scenarios with different food diversity levels by (**a**) non-RO model and (**b**) RO model.

Modeling results for the environmental impact of scaling up local food development are summarized in Figure 4 and Table 4. *Scaling up* would add at least 2.475 million m^3^ to the total irrigation water use annually. As the food diversity level increases, irrigation water for fruit and vegetable production reports a more evident increase as compared to the water use for growing livestock feeds (Figure 4). The results echo previous studies using national datasets, in which an increased intake of fruits and vegetables might contribute to larger water footprints of food consumption [31]. At a food diversity level of 17%, the modeling diet indicates that on an annual basis, a total of 10,936 m^3^ irrigation water could be required, which is about 41% of freshwater use in all sectors in the Palouse area and 153% of irrigation water currently used. Furthermore, the impact could mean 70% of freshwater being used in the sector of public supply would be needed if residents were to meet all their nutrition needs solely by consuming food grown locally.

The results of RO solutions for most of the scenarios would reflect small increases in irrigation requirements when uncertainties were accounted for (by 1–11%, Table 4). The possible reason is that strawberries and grapes, two crops that have larger water footprints as compared to that of apples, were less likely to be included by models using the non-robust optimization, but they are often selected by RO models (Figure 4). We also find that along with the increase in food diversity levels and increased consumption of fruits and vegetables, the irrigation water footprint of local foods (by weight or m^3^/1000 kg) tends to increase. Specifically, the required amount of irrigation water to produce 1000 kg of local food could rise from 22 m^3^ to 77 m^3^ (Table 4).

In addition to the impact on irrigation water use, nearly 67.59–74.90 million m^3^ of green water is needed annually if nutritional or caloric needs are satisfied 100% by local foods. Their counterparts of cropland are approximately 21 thousand hectares or only up to 5% of total cropland in the region, and more than 9 thousand hectares or about 8% of total pastureland would also be required (Table 4). Most of these resources are used to produce grains, legumes, and livestock feeds. In contrast to irrigation water, the increased level of food diversity and fruit or vegetable production might not have an evident impact on cropland, green water or pastureland use. Specifically, if the food diversity level ascends to 17% or the most diverse food production portfolio in our models, the total amount of green water would only increase by 5%, while the requirements associated with cropland and pastureland would even decline slightly by 2% and 5%, respectively. This is mostly due to the increased production of fruits and vegetables that require smaller amounts of cropland and do not require pastureland.

### 3.2. Environmental Impacts of Food Waste Reduction

As mentioned above, food waste reduction might play a proactive role in lowering environmental burdens arising from new agricultural development. We integrated the potential amounts of food grown locally and waste rates at the crop level obtained from the USDA LAFA database to determine what would be the total amount of food wasted when scaling up and its associated environmental impacts.

**Figure 5:**
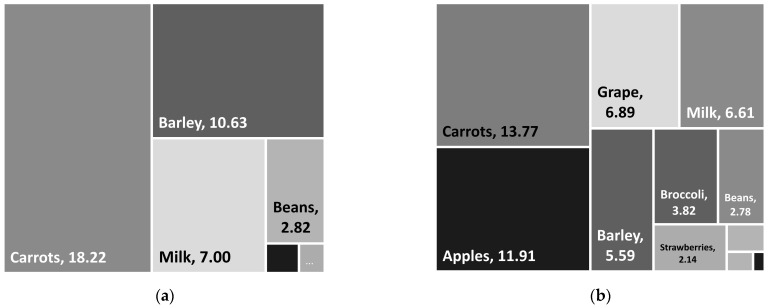
Robust-optimization estimation of potential amounts of local food wasted on the Palouse (in million kg) with (**a**) no diversity requirement and (**b**) 17% diversity level.

As shown in Table 5, for the modeling diet with 17% food diversity, up to 37% of food content (by weight) would be wasted. The rate of waste at the consumer level (excluding the inedible portion) is 24% on average, which tends to be higher than those at the retail level (14% by weight), except for fruits (see Table 5). Notably, although vegetables only account for 26% of the total production (by weight), as much as 40% of the food waste at the consumer level is from vegetables. Similarly, despite fruits only accounting for 34% of total food production, they would account for more than half of the food waste at the retail level. In general, fruits and vegetables together are suggested to contribute 71% and 70% of food waste at the retailer and consumer levels, respectively (Table 5 and Figure 5). As shown in Figure 5, when there are no explicit food diversity requirements, most food waste could be attributable to carrots (18.22 million kg), barley (10.63 million kg), and dairy products (7 million kg). However, increasing food diversity levels would lead to more food waste of fruits and vegetables in the status quo scenario. Specifically, at the food diversity level of 17%, carrots, grapes, and apples may account for more than 60% of food waste by weight (Figure 5).

Our scenario-based diet-optimization models further simulate the potential environmental benefits of food waste reduction in the context of scaling up local food development. The results indicate that, on an annual basis, if there is a 50% reduction in food waste, an average of 23 million kg of food will avoid being wasted. In other words, each resident on the Palouse would avoid wasting about 0.7 kg of food daily while still meeting their food demand. With respect to key agricultural or environmental resources, in the scenarios associated with a food diversity level of 17%, irrigation water requirements would decline from 12,151 thousand m^3^ to 9286 thousand m^3^, or by 2869 thousand m^3^ per year, which is equal to 24% of the original irrigation water requirement prior to waste reduction (Figure 5). The reduced water consumption that can be attributed to retail-level food waste reduction would be 1089 thousand m^3^ per year, and their counterparts of consumer-level food waste reduction are more evident at approximately 1800 thousand m^3^ of irrigation water per year.

**Figure 6:**
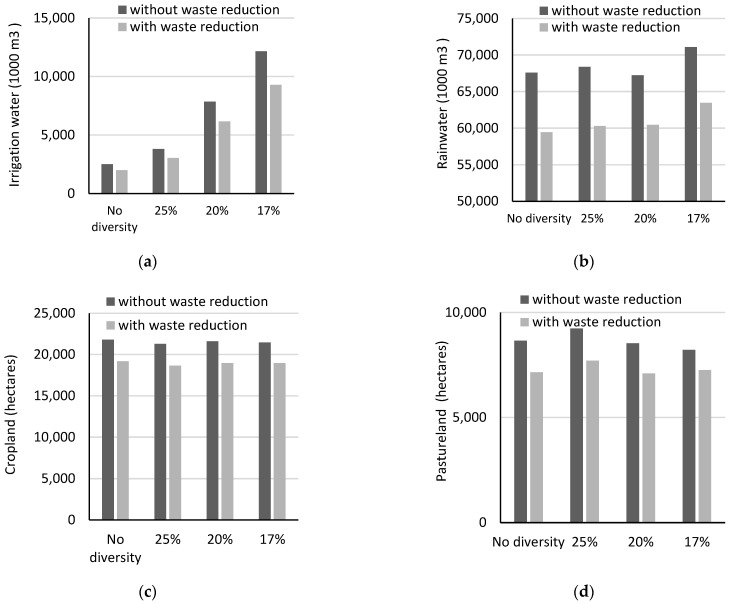
The environmental impacts of food waste reduction by (**a**) irrigation water, (**b**) rain or green water, (**c**) cropland, and (**d**) pasture.

In the same scenario, green water use may drop by approximately 11,356 thousand m^3^ per year, which is about 15% of the original amount of green water estimates (Figure 6). Similarly, 2955 hectares of cropland or 13% of the original requirement could be saved due to less farmland used to grow grains, fruits and vegetables (1067 hectares) and livestock feeds (842 hectares). Meanwhile, there is an on-average 18% reduction in pastureland requirements, and this effect mostly results from fewer land resources used to grow livestock feed in the beef and dairy sectors (1828 hectares). Furthermore, when the food diversity level increases or there is an increased consumption of fruits and vegetables, the amounts of savings across different resource types also increases, reiterating the critical role played by reducing waste in lowering the impact of scaling up on the environment (Figure 6). For example, if the food diversity level changes from 20% to 17%, the potential water savings due to reducing food waste could increase from 1692 thousand m^3^ per year or 22% of the original required amount prior to waste reduction to 2869 thousand m^3^ per year or 24% of the required amount prior to waste reduction.

## 4. Discussion

Recent studies have suggested that the growing demand for local foods and transition toward a healthier diet may be associated with location-specific resource requirements [29,35]. In our case study of the Inland Northwest Palouse region, estimates of land and water resource impacts of potential local agricultural development are as high as approximately 83 m^3^ (or more than 2100 gallons) of irrigation water, more than 500 m^3^ (or 132,000 gallons) of green water, about 1500 m^2^ (0.38 acre) of cropland, and 600 m^2^ (0.15 acre) of pastureland per 1000 kg of local food. These local-level environmental footprint metrices are generally lower than estimates of US averages using national-level datasets [29,31]. This is largely due to dryland agricultural production that requires lower amounts of blue or irrigation water for growing grains or legumes and also because our models explicitly optimize the production portfolio to find a minimum amount of irrigation water with a selected subset of food types. However, as found in other studies [20,29,51], increased food diversity levels, e.g., from no diversity to 17% food diversity, are likely to result in a substantial increase in fruit and vegetable consumption and increased amounts of irrigation water whereas the impact on other agricultural resources, including cropland, pastureland, and green water uses, would be negligible.

Our results further suggest that even though growth in small-farm production of local foods, especially fruits and vegetables, would increase the use of water and cropland resources, it would be a minor amount of the overall use and well within the range of use that can be integrated into land use and water resource management in the region. For example, to meet 10% of the theoretical food demand of those living in the Palouse region with locally grown foods, which is an aspirational goal chosen to give a sense of the scale of impacts, the expected increase in water use, i.e., 3331 m^3^ per day, remains less than 5% of the total estimated freshwater withdrawal (73,475 m^3^ per day) and less than 8% of the public-supply withdrawal. Besides irrigation, the possible impact would also be less than 1% of existing cropland and pasture, representing a tiny share (i.e., 0.62%) of the current use of green or rainfall water on the Palouse. Therefore, the environmental cost of scaling up local food development would be well within the range of potential conservation goals at the regional level.

A more vibrant local food system is by no means focused purely on food production but should include different sectors of the food system, including processing, distribution, food consumption, and waste reduction. In line with our results, substantial savings in irrigation water use and fewer acres of cropland, pastureland, and green water could be realized by food waste reduction, particularly in the consumption stage of fruits and vegetables. On the Palouse and elsewhere, several non-profit organizations, such as Food Not Bombs and Backyard Harvest, have been actively collecting surplus foods, including vegetables and fruits, from grocery stores, community and home gardens, and catered events, for redistribution to area residents at no charge. Our results provide additional numerical evidence that waste reduction in local food processing, retailing, and consumption could generate additional benefits for the local environment. Understanding and being able to quantify the potential impact of better access to local fresh produce and reducing food waste are also important as PBAC, the Palouse-Clearwater Food Coalition, municipalities, and other local agencies and organizations convene community leaders and residents to discuss and develop short- and long-term plans for water and land use within the Palouse region while moving forward for a healthier food system.

Last, the diet-optimization-based approach presented in this case study can be readily tailored to evaluate environmental influences with consideration of a variety of objectives and tradeoffs. For example, the objective of our modeling may change from minimizing irrigation water use to maximizing the total volume of food production where meat or beef consumption is explicitly required as one source of protein. In this case, the total irrigation requirement would substantially increase from 33,312 m^3^ per day to 102,698 m^3^ per day in the scenario in which 17% of the food diversity requirement is required. In the same scenario, although there was only a 1.5% increase in the total amount of foods produced, more water-intensive fruits (e.g., cherries or peaches) and vegetables (e.g., broccoli) are more likely to be selected. Along this line of inquiry, recent studies demonstrated that dairy farms run on more external inputs including herbicides, pesticides, and fertilizer, which might deteriorate the water quality in nearby rivers or streams [60]. Hence, a new scenario could be developed to evaluate the tradeoffs between minimizing the environmental impacts of dairy farming while meeting the demand for a more desirable diet for the local population. In this case, the total irrigation water requirement of scaling up would increase by 38.5% or may rise to 46,106 m^3^ per day, corresponding to an additional 63% of current freshwater use on the Palouse, despite less stress of water quality deterioration.

## 5. Conclusions

In this research, we evaluated how *scaling up* local food development, following the recommended Dietary Guidelines from USDA, would impact local water and land resources on the Palouse. We showed that a shift in agricultural production, particularly driven by increased consumption of fruits and vegetables, might not result in an evident burden on irrigation water use in the region. Furthermore, the impacts on dryland crop, pastureland, and green water uses are likely to be negligible. By modeling the effect of food waste reduction on potential agricultural resources or land and water use, we estimated that more than 35% of local foods (by mass) may be wasted in the retailing and consumption stages and that reducing food waste would help save irrigation water by up to 24% and help the region reduce the use of green water, cropland, and pastureland by approximately 13–20% while achieving substantial nutritional benefits for the local population. These results thus provide intriguing and useful information for policymakers or local food advocates to address the environmental implications of a more localized food system.

Practically, agricultural extension specialists can make use of the modeling outcomes and encourage the adoption of water-saving technologies such as drip tubes and innovative irrigation scheduling techniques that utilize soil and water sensors. It is also feasible to modify our optimization models to suit food system planning in different regions that have been faced with other environmental issues such as soil erosion, water quality, and farmland conservation. Additionally, we can customize the footprint metrics to include factors such as carbon emissions and pesticide use. However, there is also a need to better understand the advantages and limitations of utilizing footprint-based approaches for evaluating the environmental impacts of scaling up agricultural production. Furthermore, given the resource and monetary constraints of small- and medium-sized farms, additional research is also needed to better understand the economic feasibility of scaling up, especially when it is being integrated with the conventional food system (i.e., scaling over; see Brislen [8] and James [61]). It is also worth conducting research on how time discounting rates of various diets could influence people’s attempts to decrease their food waste and boost their intake of fruits and vegetables as well as to identify the underlying forces that drive cultural and behavior level changes over the longer term, which are well beyond the scope of the model [62,63]. Future scholarly efforts would be fruitful if more food types could be included in the modeling and different sources of irrigation water could be differentiated (e.g., surface and groundwater).

## Figures and Tables

**Table 1:** Agricultural and Local Food Production in the Palouse Area and the Belonging States ^1^.

	Whitman, WA	Washington	Latah, ID	Idaho
All farms				
# of farms	1039	35,793	1041	15,028
Average farm size (acre)	1240	410	336	305
Average size of farms with irrigation	56	113	3	217
Average sales per farm ($)	$268,309	$269,172	$74,901	$213,657
Vegetables and fruits				
# of farms	19	6850	41	1552
Average farm size (acre)	3.31	59.33	1.51	135.14
Average sales per farm ($)	$5737	$586,130	$5533	$276,753
Animals and livestock				
# of farms	240	14,405	325	12,305
Average farm size (acre)	612.03	256.86	55.47	351.72
Average sales per farm ($)	$79,721	$184,039	$13,606	$354,052
Farms with directly marketed retail sales				
# of farms	53	4503	72	1765
Average sales per farm ($)	$6302	$15,229	$3028	$15,865
Land use				
% of cropland irrigated	0.49	22.56	0.06	57.65
% farms with irrigated ag. land	9.80	61.43	5.99	89.96

^1^ Source: 2017 Census of Agriculture (USDA, 2019). Statewide statistics regarding vegetables are focused on operations with fresh-market sales. #: Number of farms.

**Table 2:** Estimated Use of Freshwater by Sectors on the Palouse ^1^.

Water Use (1000 m^3^/Day)	Whitman, WA		Latah, ID	
	Groundwater	Surface Water	Groundwater	Surface Water
Irrigation crops	3.75	5.75	6.93	3.07
Livestock	0.83	0.08	0.26	0.19
Irrigation golf	0.64	N/A	1.48	N/A
Public supply	21.27	N/A	21.46	N/A
Total freshwater withdrawal	27.90	5.83	36.11	3.63

^1^ Source: USGS national water estimation, 2014 [44].

**Table 3:** Water and Land Footprints of Selected Crops or Animal Products ^1^.

Food	Blue WF or Irrigation Water Requirement			Green WF	Cropland	Pasture
	County /State	Minimum	Maximum	County /State	County /State	National
	m^3^/1000 kg	m^3^/1000 kg	m^3^/1000 kg	m^3^/1000 kg	m^2^/1000 kg	m^2^/1000 kg
Wheat	-	-	-	802	2642	-
Barley	-	-	-	533	2655	-
Lentils/peas	-	-	-	948	4546	-
Beans	-	-	-	1679	5217	-
Apples	127	92	171	97	280	-
Cherries	1016	711	1320	278	1001	-
Peaches	381	267	496	278	718	-
Grapes	195	137	254	321	661	-
Strawberries	209	146	271	97	895	-
Sweet corn	232	162	302	112	569	-
Carrots	22	15	28	22	166	-
Broccoli	175	123	228	63	491	-
Tomatoes	139	97	181	55	288	-
Potato	109	76	142	24	159	-
Beef	589	401	548	14,751	8890	30,000
Chicken	205	159	217	1896	9300	-
Pork	568	385	526	3811	21,890	-
Egg	198	129	172	1836	7300	-
Turkey	221	156	205	2043	10,400	-
Milk	72	45	62	783	1290	3700

^1^ Sources: 2017 Census of Agriculture and USDA quick stats database [38,39], Marston et al. [48], Water Footprint Network, and field experiment data from the university extension.

**Table 4:** Annual agricultural resources needed for scaling up local food development and environmental footprints per 1000 kg of local food.

	Unit	No Diversity	25%	20%	17%
Non-RO model					
Irrigation	1000 m^3^	2476	3566	7181	10,936
Green water	1000 m^3^	67,592	68,383	67,236	71,094
Cropland	Hectare	21,798	21,287	21,597	21,447
Pastureland	Hectare	8658	9242	8534	8215
Irrigation	m^3^/1000 kg	22	33	58	77
Green water	m^3^/1000 kg	612	641	546	499
Cropland	m^2^/1000 kg	1974	1994	1753	1507
Pastureland	m^2^/1000 kg	784	866	693	577
RO model					
Irrigation	1000 m^3^	2502	3801	7851	12,151
Green water	1000 m^3^	67,835	68,679	69,197	74,898
Cropland	Hectare	21,833	21,219	21,673	21,913
Pastureland	Hectare	8688	9326	8614	9082
Irrigation	m^3^/1000 kg	23	35	61	83
Green water	m^3^/1000 kg	614	629	541	513
Cropland	m^2^/1000 kg	1977	1944	1694	1501
Pastureland	m^2^/1000 kg	787	855	673	622

**Table 5:** Optimal annual amount of food grown locally and associated food waste and loss ^1^.

Food Group	Amount of Food Needed	Amount of Food Wasted			
	Million kg	Retail Level		Consumer Level	
		Million kg	% of Loss	Million kg	% of Loss
Grains	14.24	1.71	12.00	3.88	27.23
Fruits	49.74	10.72	21.56	10.22	20.55
Vegetables	37.42	3.79	10.12	13.81	36.90
Meats, poultry, eggs, and dairy	24.19	2.92	12.07	4.48	18.53
Legumes (beans and peas)	20.37	1.22	6.00	1.87	9.20
Total	145.96	20.36	13.95	34.26	23.47

^1^ The estimation is based on the robust model associated with food diversity level of 17%.

## Data Availability

The data presented in this study are available on request from the corresponding author.
